# The Efficacy of Ambulatory Blood Pressure Monitoring-Derived Indices in Predicting Organ Damage in Patients With Hypertension

**DOI:** 10.7759/cureus.77457

**Published:** 2025-01-15

**Authors:** Mustafa Candemir, Betül Ayça Yamak, Hüseyin Baran Özdemir, Asife Şahinarslan

**Affiliations:** 1 Department of Cardiology, Gazi University Faculty of Medicine, Ankara, TUR; 2 Department of Cardiology, Hopa State Hospital, Artvin, TUR; 3 Department of Ophthalmology, Gazi University Faculty of Medicine, Ankara, TUR

**Keywords:** ambulatory arterial stiffness index, average real variability, blood pressure indices, hypertension-mediated organ damage, pulse pressure index

## Abstract

Background and objective

Hypertension (HT)-mediated organ damage (HMOD) refers to structural or functional damage in organs caused by chronic HT, which plays a critical role in determining cardiovascular mortality risk. Traditional ambulatory blood pressure monitoring (ABPM) parameters have limited utility in predicting its occurrence. To address this gap, we focused on indices that reflect blood pressure (BP) variability and arterial stiffness, such as average real variability (ARV), ambulatory arterial stiffness index (AASI), and pulse pressure index (PPI). This study aimed to examine the ability of these novel ABPM-derived indices to enhance the prediction of HMOD.

Methods

Seventy-nine hypertensive patients were assessed for end-organ damage in the heart, retina, and kidneys. Cardiac involvement was evaluated using echocardiography, focusing on left ventricular mass index (LVMI), relative wall thickness, and diastolic function. Patients with left ventricular hypertrophy (LVH) or diastolic dysfunction were considered to have hypertensive cardiomyopathy. Retinal damage was assessed via fundoscopic examinations, identifying changes like hemorrhages and arterial narrowing. Renal involvement was determined based on the estimated glomerular filtration rate (<60 mL/min/1.73 m²) and albuminuria (>30 mg/g). Patients were classified into HMOD and non-HMOD groups based on these criteria. ABPM was conducted to measure traditional parameters, such as 24-hour systolic BP (SBP) and diastolic BP (DBP), and novel indices like ARV, AASI, and PPI. Regression analysis, including demographic characteristics such as age and gender, was also used to evaluate the independent predictive value of ARV, AASI, and PPI.

Results

Traditional ABPM parameters, including 24-hour BP, did not significantly differ between patients with and without HMOD. However, novel indices such as ARV, AASI, and PPI were significantly higher in the HMOD group and showed stronger predictive value. ARV (OR: 2.026, 95% CI: 1.294-3.171, p=0.002), AASI (OR: 4.950, 95% CI: 1.840-13.317, p=0.002), and PPI (OR: 1.209, 95% CI: 1.081-1.352, p=0.001) emerged as independent predictors of HMOD.

Conclusions

ARV, AASI, and PPI were found to independently predict HMOD, providing a useful tool for better risk assessment in hypertensive patients. These results suggest that novel ABPM indices could improve early detection and management of HMOD. Further research involving larger and more diverse populations is needed to confirm these findings and guide clinical use.

## Introduction

Hypertension (HT) is one of the leading causes of morbidity and mortality globally, contributing to over 10 million deaths annually, primarily due to cardiovascular and renal complications [1]. Although the global age-adjusted rates of HT have stayed relatively unchanged over the past 30 years (affecting about 32% of women and 34% of men), the number of people diagnosed with HT has nearly doubled. This surge is attributed mainly to the growing proportion of older adults in the overall population [2]. Its prevalence varies regionally, driven by factors such as socioeconomic status, healthcare access, and lifestyle trends, with higher rates observed in Central and Eastern Europe, Central Asia, and parts of Latin America [2]. HT prevalence in Turkey aligns with global trends, affecting approximately one-third of the adult population [3].

Long-standing elevated blood pressure (BP) can damage the arteries and end organs, such as the kidneys, brain, and heart. Although this damage may initially be asymptomatic, it can result in overt cardiovascular events over time, especially if left untreated [4]. The presence of HT-mediated organ damage (HMOD) increases cardiovascular risk in patients [4]. Recognizing and understanding HMOD is essential, as it provides a critical opportunity to identify high-risk individuals early and tailor treatment strategies to reduce long-term complications.

Clinic-based measurements are often insufficient for diagnosing conditions such as masked HT, which is strongly associated with increased cardiovascular risk [5], and white coat HT, which may pose a lower risk but still requires careful monitoring due to its potential to progress to sustained hypertension [6,7]. Ambulatory blood pressure monitoring (ABPM) offers valuable data for diagnosing and monitoring patients with HT. Unlike traditional clinic-based measurements that capture only a single reading at a specific point in time, ABPM offers continuous monitoring over 24 hours, providing a more comprehensive view of BP variability. It also enables the detection of diurnal patterns, such as day-night fluctuations and morning surges, as well as abnormal profiles like non-dipping, all of which are strongly linked to cardiovascular events [8]. While systolic BP (SBP) and diastolic BP (DBP) measurements were the primary indicators used to assess cardiovascular risk in both hypertensive and normotensive individuals for many years, these measurements only represent the highest and lowest points in the more complex BP cycle [9].

Studies have introduced various parameters, such as the pulse pressure index (PPI) [10], ambulatory arterial stiffness index (AASI) [11], and average real variability index (ARV) [12], to improve the assessment of BP and cardiovascular risk. PPI reflects arterial compliance by representing the ratio of pulse pressure to SBP. AASI evaluates arterial stiffness through the regression slope of DBP on SBP over 24 hours. ARV measures short-term BP variability, capturing fluctuations missed by single-point measurements. Unlike traditional BP measurements, which focus on static systolic and diastolic values at specific moments, these parameters provide dynamic insights into vascular health and BP variability, highlighting their potential role in understanding the mechanisms of arterial stiffness and endothelial dysfunction. These changes contribute to HMOD by elevating vascular resistance and exacerbating organ damage [13].

Previous studies have associated these parameters with cardiovascular outcomes [14,15] and highlighted their role in evaluating arterial stiffness and endothelial dysfunction [13,16]. However, their specific application in the context of HMOD remains relatively limited. This study aims to address gaps in previous research by evaluating the specific role of ABPM-derived parameters, such as PPI, AASI, and ARV, in the context of HMOD. The findings could have significant clinical implications, as these parameters may enhance risk stratification and enable earlier detection of hypertensive patients at high risk for organ damage. This, in turn, could guide more personalized management strategies, potentially improving patient outcomes and preventing the progression of HT-mediated complications. The study aims to evaluate the predictive value of ABPM-derived parameters - PPI, AASI, and ARV - in detecting HMOD.

## Materials and methods

Study design and population

We employed a cross-sectional study design to assess the relationship between HMOD and ABPM parameters. The study included 79 patients who visited cardiology outpatient clinics from February 2022 to February 2023 and met the eligibility criteria. Each participant underwent 24-hour ABPM and was evaluated for HMOD. Using an effect size of 0.5, a power of 80%, and a significance level of 0.05, a minimum of 75 participants was required for the study.

Patients included in the study either had an existing diagnosis of primary HT or were newly diagnosed based on the European Society of Cardiology guidelines [17]. Secondary HT was systematically excluded through medical history, laboratory evaluations, and imaging studies where necessary. Hypertensive patients were further categorized into two subgroups, depending on whether they exhibited HMOD. Patients were excluded from the study if they had acute coronary syndrome, acute heart failure, congenital heart defects, severe valvular heart conditions, atrial fibrillation, diabetes, morbid obesity, asthma or chronic obstructive pulmonary disease, psychiatric or neurological disorders, endocrine conditions, alcohol or substance dependence, acute infections, or connective tissue disorders. In addition, patients with chronic conditions such as chronic heart failure and chronic coronary syndromes were excluded to prevent their potential influence on ABPM parameters.

This study adhered to ethical guidelines and principles outlined in the Declaration of Helsinki. Ethical approval was obtained from the Gazi University Ethical Review Board (Approval Number: 2024-1418). All participants provided written informed consent before enrollment in the study. Participants were fully informed about the study's purpose, procedures, and their right to withdraw at any time without prejudice.

Data collection

Laboratory Assessments

Laboratory evaluations for the study participants included complete blood count, kidney and liver function tests, fasting blood glucose levels, lipid profiles, and albumin-to-creatinine ratio (ACR) in spot urine samples. Blood samples were collected, processed, and stored appropriately for subsequent analyses.

ABPM

ABPM was performed using a validated device (GE Healthcare Tonoport V, Berlin, Germany). Calibration of the ABPM devices was verified before use to ensure accurate readings. Patients were instructed to follow specific guidelines during the ABPM monitoring period, such as maintaining usual daily activities, avoiding excessive physical exertion, and refraining from consuming caffeine or alcohol, to reduce variability. The monitoring period began between 08:00 and 10:00 and continued for 24 hours. The device was programmed to measure BP every 20 minutes during the day (06:00 to 21:59) and every 30 minutes at night (22:00 to 05:59). To ensure accurate results, at least 70% of the measurements had to be valid, with a minimum of 20 readings during the day (at least two per hour) and at least seven readings at night (at least one per hour). Diagnostic criteria for HT were based on average daytime, nighttime, and 24-hour BP values: daytime SBP/DBP equal to or above 135/85 mmHg, nighttime SBP/DBP equal to or above 120/70 mm Hg, or a 24-hour mean SBP/DBP equal to or above 130/80 mmHg [17].

The study analyzed several ABPM parameters, including traditional measures, such as the average SBP and DBP for daytime and nighttime periods and the 24-hour average of SBP and DBP, along with additional metrics not typically used in routine practice. PPI was calculated as the ratio of pulse pressure (difference between SBP and DBP) to SBP [10]. AASI was defined as 1 minus the regression slope of DBP on SBP during the 24-hour monitoring period [11]. ARV was calculated as the average of the absolute value changes between consecutive BP measurements [12].

Diagnostic criteria for HMOD

Hypertensive patients were assessed for end-organ damage, specifically in the heart, retina, and kidneys. They were subsequently categorized into two groups: those with and without HMOD. Cardiac damage, defined as hypertensive cardiomyopathy, was detected using echocardiography with a Vivid 7 Digital ultrasound device (GE Healthcare, Horten, Norway) to assess left ventricular structure and function. Patients with left ventricular hypertrophy (LVH) or diastolic dysfunction were considered to have hypertensive heart disease [18]. The left ventricular mass index (LVMI) is determined by dividing the left ventricular mass by the body surface area of the patient. Concentric LVH is identified when the relative wall thickness, which represents the ratio of the thickness of the ventricular wall to the size of the chamber, exceeds 0.43. LVH is diagnosed when the LVMI exceeds 115 g/m² in men and 95 g/m² in women [17]. To ensure that the LVH analyzed in this study was primarily caused by HT, patients with conditions known to independently cause LVH, such as hypertrophic cardiomyopathy, obesity-associated LVH, chronic anemia, and hepatitis C infection, were excluded. Exclusions were based on clinical history, laboratory findings, and echocardiographic parameters consistent with nonhypertensive causes of LVH.

Diastolic function was evaluated following standard clinical guidelines using key echocardiographic parameters. These included mitral inflow velocities (E and A waves), the E/A ratio, tissue Doppler imaging to measure e' velocity, and the E/e' ratio to estimate left ventricular filling pressures. Additional parameters, such as the left atrial volume index (LAVI) >34 mL/m² and tricuspid regurgitation velocity (TRV) >2.8 m/s, were also considered. Based on these measurements, diastolic dysfunction was classified into different grades to assess the degree of impaired ventricular filling [19]. Grade I (impaired relaxation) was defined by an E/A ratio <0.8. For Grades II (pseudonormal filling) and III (restrictive filling), additional criteria were considered, including an E/A ratio between 0.8-2.0 and >2.0, respectively, along with evidence of elevated filling pressures. An E/e' ratio >14, LAVI >34 mL/m², and TRV >2.8 m/s were critical in grading diastolic dysfunction. Additionally, septal e' velocity <7 cm/s and lateral e' velocity <10 cm/s were considered significant when combined with other indices, such as elevated TRV or LAVI.

For hypertensive retinopathy, fundoscopic examinations were conducted to detect retinal changes such as hemorrhages, arterial narrowing, and optic disc abnormalities. We diagnosed hypertensive nephropathy if the estimated glomerular filtration rate (eGFR) was below 60 mL/min/1.73 m² or albuminuria exceeded 30 mg/g [8]. The eGFR was calculated using the CKD-EPI (Chronic Kidney Disease Epidemiology Collaboration) formula.

Inter-rater reliability for echocardiographic parameters was evaluated using Cohen's kappa coefficient, which demonstrated strong agreement between raters with a value of 0.91. Similarly, intra-observer reliability for fundoscopic examinations was assessed, yielding a Cohen's kappa value of 0.94, indicating excellent consistency between repeated measurements.

Statistical analysis 

All data were analyzed using SPSS Statistics version 25.0 (IBM, Armonk, NY). Continuous variables were presented as mean ± standard deviation (SD) or median [interquartile range (IQR)] according to the distribution pattern, and categorical data were expressed as percentages or frequencies. Continuous variables were examined using the Kolmogorov-Smirnov test to check for normal distribution. Clinical features and laboratory measurements were compared between groups using the Student’s t-test, Mann-Whitney U test, or the chi-square test. In the pairwise comparison tests, regression analysis was performed to evaluate potential confounders. Multivariable logistic regression analyses were performed to determine the independent predictors of HMOD in patients with HT. Variables with p<0.25 in univariable logistic regression and believed to be of clinical importance were included in a multivariable logistic regression model.

Various methods were employed to resolve any potential confounders in the regression analysis. Firstly, our study had no missing data or outliers. Outliers in the numerical values were assessed using residual statistics, with standardized residuals within the range of -329 to +329 and Cook’s distance <0.5 for all parameters. For the regression analysis, numerical data were standardized. Additionally, to assess multicollinearity, Pearson correlation, tolerance values, variance inflation factor (VIF), and condition indices were examined. No multicollinearity was found among the independent variables, as indicated by Pearson correlation coefficients (r) <0.70, tolerance values >0.25, VIF <4, and condition number <10 for all parameters. A two-tailed p-value ≤0.05 indicates that the related parameter is statistically significant.

## Results

**Figure 1:**
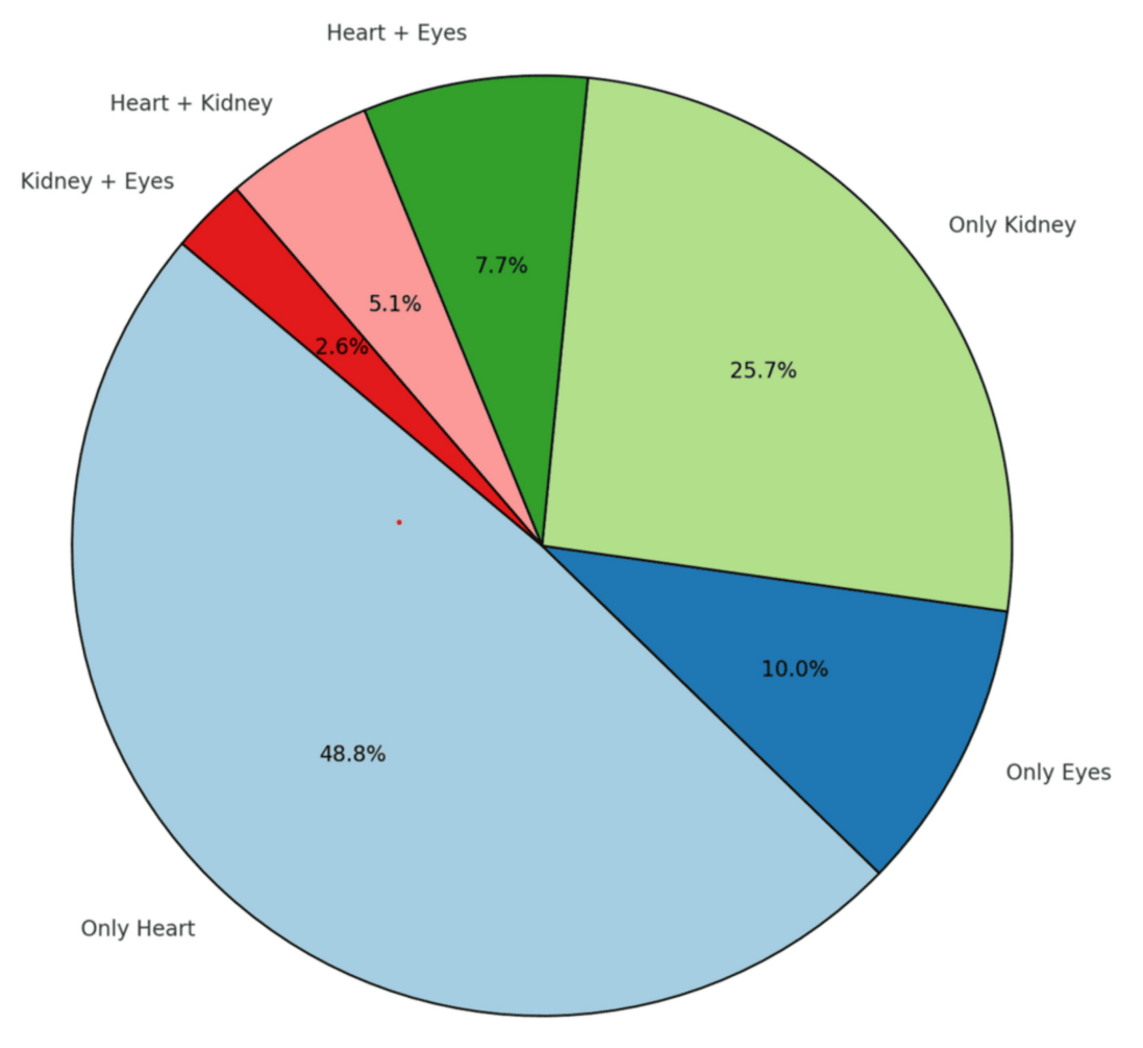
Proportional distribution of HMOD types HMOD: hypertension-mediated organ damage

The study included 79 patients with HT, a mean age of 50 ± 12 years. Among them, 47% were female, and 53% were male. HMOD was detected in 39 patients (Table 1). Upon evaluating HMOD, it was found that the cardiac system was the most commonly affected, whereas the ocular system was the least affected. No patients exhibited involvement of all three organs simultaneously. However, when examining organ co-involvement, a combination of heart and eye involvement was observed (Figure 1).

**Table 1:** HMOD types in patients with hypertension HMOD: hypertension-mediated organ damage

Organ damage type	Number of patients with HMOD	Percentage of total HMOD cases
Only heart	19	48.7
Only eyes	4	10
Only kidney	10	25.6
Heart + eyes	3	7.7
Heart + kidney	2	5.1
Kidney + eyes	1	2.6
Heart + eyes + kidney	0	0
Total HMOD cases	39	100

Table 2 summarizes the demographic and laboratory characteristics of HT patients with and without HMOD. BMI was lower in the HMOD group (p=0.027). Renal involvement was reflected in significantly higher levels of urine microalbumin, protein, and albumin/creatinine ratios in the HMOD group (p<0.001, p=0.038, and p<0.001, respectively). There were no statistically significant differences between patients with and without HMOD regarding age, gender, smoking, hemoglobin, GFR, and urine creatinine (p>0.05 for all parameters).

**Table 2:** Demographics, clinical features, and laboratory measurements in patients with hypertension Statistically significant results (p<0.05) are shown in bold BMI: body mass index; BUN: blood urea nitrogen; GFR: glomerular filtration rate; HbA1c: hemoglobin A1c; HMOD: hypertension-mediated organ damage; HT: hypertension; IQR: interquartile range; SD: standard deviation; TSH: thyroid-stimulating hormone; WBC: white blood cells

Variable	HT with HMOD (n=39)	HT without HMOD (n=40)	P-value
Age, years, mean ± SD	50.1 ± 12.3	49.7 ± 11.3	0.881
Male gender, n (%)	22 (56.4)	16 (40)	0.144
BMI, kg/m^2^, mean ± SD	27.75 ± 4.77	30.04 ± 4.24	0.027
Smoking, n (%)	14 (35.9)	13 (32.5)	0.750
Hemoglobin, g/dl, mean ± SD	14.57 ± 1.48	14.17 ± 1.6	0.247
WBC count, x10³/mm³, mean ± SD	7.72 ± 2.11	7.6 ± 1.9	0.836
Fasting blood sugar, mg/dl, median (IQR)	94 (84–103)	93 (84–106)	0.746
HbA1c, %, median (IQR)	5.8 (5.4–6.1)	5.7 (5.4–6.2)	0.798
BUN, mg/dl, mean ± SD	15.17 ± 4.47	13.93 ± 3.43	0.168
Creatinine, mg/dl, mean ± SD	0.79 ± 0.15	0.75 ± 0.14	0.230
GFR, ml/min/1.73m², mean ± SD	87.56 ± 5.83	87.60 ± 4.98	0.977
Uric acid, mg/dl, median (IQR)	5.60 (4.60–7)	5.20 (4.30–6.35)	0.359
Sodium, mmol/L, mean ± SD	140.18 ± 1.73	140.28 ± 1.89	0.816
Potassium, mmol/L, mean ± SD	4.32 ± 0.38	4.35 ± 0.34	0.778
Total protein, g/dl, mean ± SD	7.36 ± 0.40	7.29 ± 0.42	0.450
Albumin, g/dl, mean ± SD	4.44 ± 0.26	4.47 ± 0.24	0.552
Calcium, mg/dl, mean ± SD	9.91 ± 1.74	9.54 ± 0.46	0.206
TSH, mIU/L, median (IQR)	1.75 (1.29–2.80)	1.86 (1.04–2.57)	0.666
Urine microalbumin, mg/dl, median (IQR)	12.29 (1.36–81.08)	0.95 (0.50–1.69)	<0.001
Urine protein, mg/dl, median (IQR)	10.77 (6.90–22)	7.49 (6.18–11.57)	0.038
Urine creatinine, mg/dl, median (IQR)	100.40 (67.21–158.26)	107.16 (60.65–146.33)	0.837
Urine protein/creatinine ratio, mg/g, median (IQR)	0.10 (0.06–0.18)	0.07 (0.05–0.11)	0.012
Urine albumin/creatinine ratio, mg/g, median (IQR)	19.17 (10.87–43.07)	9.04 (4.80–13.12)	<0.001

The study revealed significant echocardiographic differences between hypertensive patients with and without HMOD. Interventricular septum thickness (IWST) and posterior wall thickness (PWT) were significantly higher in the HMOD group (p<0.001; p=0.001, respectively). Relative wall thickness (RWT) was also elevated in the HMOD group (p<0.001). The HMOD group had a significantly higher LVMI (p=0.003), reflecting hypertensive-induced cardiac remodeling and LVH. Additionally, the HMOD group exhibited significantly higher TRV (p<0.001) and E/e’ ratio (p<0.001), indicating marked differences in diastolic function and pulmonary pressures. There were no statistically significant differences in diastolic/systolic diameters, ejection fraction, left atrium-related parameters, and the E/A ratio between hypertensive patients with and without HMOD (p>0.05 for all parameters) (Table 3).

**Table 3:** Transthoracic echocardiographic parameters in hypertensive patients with and without HMOD Statistically significant results (p<0.05) were shown in bold DT: deceleration time; HMOD: hypertension-mediated organ damage; HT: hypertension; IQR: interquartile range; IVST: interventricular septum thickness; LA: left atrium; LAVI: left atrium volume index; LVED: left ventricular end-diastolic diameter; LVEF: left ventricular ejection fraction; LVES: left ventricular end-systolic diameter; LVM: left ventricular mass; LVMI: left ventricular mass index; PWT: posterior wall thickness; RWT: relative wall thickness; SD: standard deviation; TRV: tricuspid regurgitation velocity

Echocardiographic parameter	HT with HMOD (n=39)	HT without HMOD (n=40)	P-value
Aorta, cm, mean ± SD	28.38 ± 2.94	28.15 ± 2.42	0.700
Ascending aorta, cm, mean ± SD	34.41 ± 3.41	34.43 ± 3.32	0.985
LVED, cm, mean ± SD	4.54 ± 0.41	4.52 ± 0.40	0.862
LVES, cm, mean ± SD	2.76 ± 0.43	2.87 ± 0.41	0.241
IVST, cm, median (IQR)	1.20 (1.10–1.30)	1.05 (1.00–1.10)	<0.001
PWT, cm, median (IQR)	1.10 (1.00–1.20)	1.00 (0.90–1.06)	0.001
RWT, mean ± SD	0.49 ± 0.10	0.42 ± 0.04	<0.001
LVEF, %, mean ± SD	64.26 ± 4.67	64.70 ± 5.12	0.689
LA, cm, mean ± SD	3.67 ± 0.43	3.77 ± 0.40	0.314
LA volume, ml, mean ± SD	58.09 ± 15.36	51.75 ± 15.85	0.075
LAVI, ml/m², median (IQR)	26.96 (20.90–31.42)	26.27 (22.02–31.29)	0.984
E/A ratio, median (IQR)	0.90 (0.80–1.30)	0.99 (0.73–1.25)	0.902
DT, msec, median (IQR)	225 (190–257)	202 (183.50–229)	0.255
TRV, m/sn, mean ± SD	2.49 ± 0.60	1.95 ± 0.45	<0.001
Septal e’, cm/s, median (IQR)	6 (5–8)	7.50 (6–9)	0.012
Lateral e’, cm/s, median (IQR)	9 (7–11)	11.50 (8.25–14)	0.007
E/e’ ratio, median (IQR)	11 (8.15–16)	7 (6–8)	<0.001
LVM, g, median (IQR)	179.96 (136.20–203.05)	155.71 (132.18–184.75)	0.077
LVMI, g/m², median (IQR)	92.34 (80–113.23)	79.73 (67.35–93.89)	0.003

There was no difference in traditional ABPM parameters between the HMOD group and the non-HMOD group. However, the HMOD group had significantly higher ARV, AASI, and PPI values (p<0.001 for all). There were no statistically significant differences in daytime, nighttime, or 24-hour systolic and diastolic blood pressures, and diastolic/systolic load values between hypertensive patients with and without HMOD (p>0.05 for all parameters) (Table 4).

**Table 4:** ABPM parameters in patients with hypertension Statistically significant results (p<0.05) were shown in bold AASI: ambulatory arterial stiffness index; ABPM: ambulatory blood pressure monitoring; ARV: average real variability; DBP: diastolic blood pressure; HMOD: hypertension-mediated organ damage; IQR: interquartile range; PPI: pulse pressure index; SBP: systolic blood pressure; SD: standard deviation

Parameter	HT with HMOD (n=39)	HT without HMOD (n=40)	P-value
24-h SBP, mmHg, mean ± SD	136.82 ± 17.57	137.58 ± 13.91	0.833
24-h DBP, mmHg, median (IQR)	81 (73–89)	79 (73–87.75)	0.432
Daytime SBP, mmHg, mean ± SD	140.74 ± 17.63	140.88 ± 14.39	0.971
Daytime DBP, mmHg, mean ± SD	85.31 ± 11.84	82.58 ± 9.77	0.266
Night-time SBP, mmHg, mean ± SD	129.91 ± 18.65	129.10 ± 15.36	0.978
Night-time DBP, mmHg, mean ± SD	74.36 ± 10.67	73.25 ± 10.07	0.638
24-h systolic load, %, median (IQR)	41 (16–77)	47 (16.25–68.75)	0.803
Daytime systolic load, %, median (IQR)	38 (4–68)	36 (12–67.75)	0.712
Night-time systolic load, %, median (IQR)	58 (17–100)	62.50 (36–92)	0.898
24-h diastolic load, %, median (IQR)	20 (8–56)	19 (5.25–49.50)	0.613
Daytime diastolic load, %, median (IQR)	20 (4–58)	14 (3.25–45.25)	0.349
Night-time diastolic load, %, median (IQR)	25 (8–58)	31.50 (8.25–58)	0.708
Nocturnal SBP reduction, %, median (IQR)	8.60 (4.70–11.70)	9.70 (3.55–13.45)	0.677
Nocturnal DBP reduction, %, median (IQR)	12.30 (8.60–19.10)	11.30 (6.12–18.35)	0.336
ARV, mean ± SD	14.08 ± 2.49	11.93 ± 1.58	<0.001
AASI, mean ± SD	0.69 ± 0.12	0.58 ± 0.08	<0.001
PPI, mean ± SD	58.54 ± 11.34	48.07 ± 6.68	<0.001

**Figure 2:**
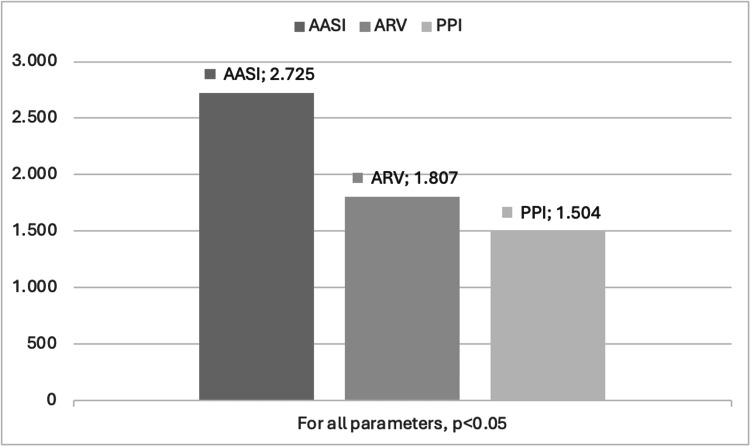
Graphical representation of the odds ratios of statistically significant parameters obtained from binary regression analysis for HMOD AASI: ambulatory arterial stiffness index; ARV: average real variability; HMOD: hypertension-mediated organ damage; PPI: pulse pressure index

Table 5 presents the logistic regression analysis results for predicting HMOD. In the univariate analysis, variables such as BMI, ARV, AASI, and PPI showed significant associations with HMOD (p<0.05). However, in the multivariable regression model, after adjusting for confounders, ARV (OR: 1.807, 95% CI: 1.081-4.150, p=0.003), AASI (OR: 2.725, 95% CI: 1.303-6.232, p=0.003), PPI (OR: 1.504, 95% CI: 1.050-2.465, p=0.002), and gender (OR: 0.210, 95% CI: 0.049-0.906, p=0.036) remained independent predictors. Other variables, including age, BMI, smoking, and HbA1c, were not significantly associated with HMOD (p>0.05). Figure 2 presents the graphical representation of odds ratios for the statistically significant predictors of HMOD obtained from binary regression analysis.

**Table 5:** Logistic regression analysis of factors predicting HMOD in hypertension patients Significant p-values are shown in bold Hosmer-Lemeshow test p-value: 0.910. Nagelkerke R²: 0.643 AASI: ambulatory arterial stiffness index; ARV: average real variability; BMI: body mass index; HbA1c: hemoglobin A1c; LVEF: left ventricle ejection fraction; HMOD: hypertension-mediated organ damage; PPI: pulse pressure index

Variable	Univariate analysis			Multivariable analysis		
	Odds ratio	95% confidence interval (lower–upper)	P-value	Odds ratio	95% confidence interval (lower–upper)	P-value
Age	1.042	0.688–1.625	0.855	-	-	-
Gender	0.515	0.211–1.261	0.146	0.21	0.049–0.906	0.036
BMI	0.581	0.353–0.956	0.033	1.034	0.996–1.079	0.063
Smoking	1.163	0.459–2.949	0.75	-	-	-
HbA1c	1.025	0.481–2.182	0.949	-	-	-
LVEF	0.981	0.896–1.075	0.685	-	-	-
ARV	1.637	1.260–2.126	<0.001	1.807	1.081–4.150	0.003
AASI	2.586	1.542–4.337	<0.001	2.725	1.303–6.232	0.003
PPI	1.143	1.066–1.224	<0.001	1.504	1.050–2.465	0.002

Table 6 shows the results of the receiver operating characteristic (ROC) analysis for the AASI, ARV, and PPI in predicting HMOD in patients with HT. The area under the curve (AUC) values for AASI, ARV, and PPI were 0.744, 0.744, and 0.772, respectively, indicating good diagnostic performance for all three biomarkers. The 95% confidence intervals for AASI (0.636-0.852), ARV (0.636-0.852), and PPI (0.670-0.875) did not include 0.5, confirming the reliability of these biomarkers in predicting HMOD. All parameters showed statistically significant results (p<0.001. The cut-off values for predicting HMOD were 0.627 for AASI, 12.525 for ARV, and 51.818 for PPI. These values were crucial for clinical application, providing thresholds for risk classification. In terms of sensitivity and specificity, PPI exhibited the highest values with 0.718 sensitivity and 0.700 specificity, followed by AASI with 0.667 sensitivity and 0.650 specificity, and ARV with 0.667 sensitivity and 0.625 specificity. These findings suggest that all three parameters have moderate to good sensitivity and specificity in identifying patients at risk for HMOD.

**Table 6:** ROC analysis results of AASI, ARV, and PPI to predict HMOD in patients with HT Significant p-values are shown in bold AASI: ambulatory arterial stiffness index; ARV: average real variability; AUC: Area under the curve; CI: confidence interval; HMOD: hypertension-mediated organ damage; PPI: pulse pressure index; ROC: receiver operating characteristic

Index	AUC	95% CI lower	95% CI upper	P-value	Cut-off value	Sensitivity	Specificity
AASI	0.744	0.636	0.852	<0.001	0.627	0.667	0.650
ARV	0.744	0.636	0.852	<0.001	12.525	0.667	0.625
PPI	0.772	0.670	0.875	<0.001	51.818	0.718	0.700

## Discussion

Our findings showed that while traditional ABPM parameters did not significantly differ between hypertensive patients with and without HMOD, significant differences were observed in less commonly used parameters such as ARV, AASI, and PPI. Additionally, logistic regression analysis revealed that gender, ARV, AASI, and PPI were significant predictors of HMOD. Although the literature indicates that the risk of HMOD is higher with increased cardiovascular risk factors such as smoking, BMI, and advancing age [4], our study did not identify a significant association between these factors and HMOD in hypertensive patients. This discrepancy may be attributed to several factors, including differences in the sample population, study design, or unmeasured confounders. Additionally, the effect of these risk factors on HMOD might be more pronounced in a larger or more diverse cohort, or with longer follow-up periods, which could better capture their impact.

Echocardiographic findings revealed significant differences between hypertensive patients with and without HMOD, particularly regarding cardiac remodeling and diastolic function markers. IVST, PWT, and RWT were higher in the HMOD group, indicating early hypertensive heart disease. LVMI was also higher, reflecting hypertensive-induced cardiac remodeling. Another cardiac consequence of HT, especially in patients who develop LVH, is diastolic dysfunction. Diastolic dysfunction is common in hypertensive patients, with its prevalence varying widely [20]. While the E/A ratio has been historically used to assess diastolic dysfunction, its utility is limited [21].

In our study, no significant differences were observed in the E/A ratio between groups, suggesting limitations in detecting subtle diastolic dysfunction. However, TRV and E/e' ratio were significantly elevated in the HMOD group, indicating impaired diastolic function and elevated pulmonary pressures. TRV reflects increased right ventricular afterload, and the E/e' ratio is a strong marker of elevated left ventricular filling pressures. These findings align with the SPHERE study by Dini et al., which showed that the E/e' ratio is a valuable marker for diastolic dysfunction in hypertensive patients [22]. An elevated TRV is associated with pulmonary HT and elevated right ventricular afterload, which can result from chronic HT and remodeling of the pulmonary vasculature [23]. Although LAVI is a known indicator of left atrial remodeling, no significant difference was found between groups in our study. LAVI measurement can be influenced by various factors, and it may increase over time as hypertensive heart disease progresses. Further studies are needed to explore LAVI's role in HMOD progression.

The capacity of non-traditional ABPM variables such as PPI, ARV, and AASI to predict mortality and morbidity has gained importance in recent years, leading to new findings that reinforce the clinical value of these parameters. These indices are emerging as valuable tools in clinical practice, as they offer insights into blood pressure variability and vascular compliance, which are more closely linked to the pathophysiological mechanisms underlying HMOD than traditional parameters such as SBP or DBP. The use of PPI has been suggested to overcome the limitations of pulse pressure in predicting cardiovascular events [10], as it reflects both vessel stiffness and dynamic compliance. In our study, PPI values were significantly higher in the HMOD group, suggesting its potential role in identifying organ damage.

Furthermore, ARV was significantly elevated in HMOD patients, indicating that increased blood pressure variability may promote endothelial dysfunction, contributing to HMOD [24]. AASI, a novel measure of vascular stiffness derived from ABPM, has been shown to predict cardiovascular mortality and is associated with subclinical organ damage, including microalbuminuria, LVH, and carotid atherosclerosis [25,26]. Studies by Hansen et al. and Dolan et al. have highlighted the association of AASI and ARV with cardiovascular mortality, particularly stroke mortality, further reinforcing the clinical relevance of these non-traditional ABPM indices in predicting long-term cardiovascular risk [14,25].

One potential explanation for the lack of significance for traditional ABPM parameters, such as SBP or DBP, could be that these measurements capture static blood pressure values and fail to reflect the dynamic fluctuations or vascular stiffness that contribute to hypertensive organ damage. ARV, AASI, and PPI, on the other hand, provide insights into blood pressure variability and arterial compliance, which are more closely linked to the pathophysiological processes underlying HMOD. HMOD is a complex process where arterial stiffness and blood pressure variability play crucial roles [27,28]. Given the complexity of these processes, it is evident that using indices like ARV, PPI, and AASI, which reflect these intricate mechanisms, provides a better assessment of organ damage in hypertensive patients compared to traditional ABPM parameters.

Differences in study populations, sample sizes, methodologies, and baseline treatment with antihypertensive medications may account for some of the discrepancies observed in the literature. While the studies mentioned focused on specific organ systems, our study assessed a broader range of hypertensive organ damage, including the heart, kidneys, and eyes. Additionally, while those studies primarily compared ABPM with clinic measurements, our research expands on the significance of non-traditional ABPM variables in predicting HMOD. These findings suggest that ARV, AASI, and PPI may be most beneficial in hypertensive patients with suspected subclinical organ damage or significant blood pressure variability, where traditional ABPM parameters may not provide sufficient diagnostic value. However, further research is needed to validate these indices in larger and more diverse populations to confirm their generalizability. Additionally, their alignment with existing HT management guidelines must be assessed to determine their practical integration into routine clinical practice. The wide confidence intervals for AASI and ARV may be due to the relatively small sample size, while the literature review shows similar confidence intervals for both AASI and ARV values [29].

This study has several limitations. Although the findings are significant and provide valuable insights for future research, the small sample size of our study reduces its statistical power. Additionally, as our study was conducted in a specific region, its generalizability may be limited. Furthermore, while our study was not age-dependent, conducting it in either a younger or older population could have yielded different results. The presence of comorbidities or increased medication use in older age groups may have influenced the outcomes. It should also be noted that echocardiographic findings may vary with age, especially in older individuals. Many factors could have influenced the results, and these could have been better understood by analyzing them separately while adjusting for age, gender, and other variables.

To account for these effects and minimize the confounding impact, the regression analysis results, conducted to mitigate these influences, should be emphasized. Moreover, patients being on different antihypertensive treatments may have affected the results, as medications can influence ABPM parameters such as AASI and ARV in varying ways. Future analyses should incorporate multivariable adjustments to confirm the independence of these associations. Additionally, differences in treatment adherence and duration were not controlled for.

## Conclusions

The aim of this study was to compare traditional and non-traditional ABPM parameters in predicting HMOD and to evaluate the clinical utility of ARV, AASI, and PPI in hypertensive patients. Our findings indicate that ARV, AASI, and PPI are independent predictors of HMOD in hypertensive patients, providing valuable insights into blood pressure variability and arterial compliance. These indices were found to be more effective in identifying organ damage than traditional blood pressure measurements. The use of these non-traditional ABPM parameters in clinical practice could aid in the early detection and better management of patients at risk for HMOD, potentially improving patient outcomes and guiding treatment strategies.
